# Functional Characterization of *TNFα* in the Starry Flounder (*Platichthys stellatus*) and Its Potential as an Immunostimulant

**DOI:** 10.3390/ani15142119

**Published:** 2025-07-17

**Authors:** Min-Young Sohn, Gyoungsik Kang, Kyung-Ho Kim, Ha-Jeong Son, Chan-Il Park

**Affiliations:** 1 Department of Marine Biology & Aquaculture, College of Marine Science, Gyeongsang National University, Tongyeong 53064, Republic of Korea; thisdancemoment@naver.com (M.-Y.S.); jemma9120@naver.com (H.-J.S.); 2 Department of Aquatic Life Medicine, College of Marine Science, Gyeongsang National University, Tongyeong 53064, Republic of Korea; gyoungsikkang@gmail.com (G.K.); rsiv94@naver.com (K.-H.K.)

**Keywords:** *TNFα*, molecular adjuvant, starry flounder

## Abstract

*Tumor necrosis factor alpha* (*TNFα*) is a central pro-inflammatory cytokine that mediates host immune responses during infection. In this study, we identified and characterized the *TNFα* gene in the starry flounder (*Platichthys stellatus*) through transcriptomic analysis. The deduced protein contained a conserved TNF domain and transmembrane region, and phylogenetic analysis confirmed its homology with other teleost TNFα proteins. Tissue-specific expression profiling revealed high baseline expression in immune-related peripheral organs and a distinct temporal modulation in response to *Streptococcus parauberis* infection. Recombinant TNFα (rTNFα), produced using a cell-free expression system, significantly enhanced phagocytic activity in peripheral and kidney-derived leukocytes in a dose-dependent manner. Peak activity was observed at 150–200 μg/mL, while a decline at higher concentrations suggested a threshold for immune stimulation. Importantly, hemolysis assays confirmed the safety of rTNFα even at the highest tested concentrations. These results demonstrate the immunomodulatory potential of TNFα as a molecular adjuvant in aquaculture vaccines and underscore its potential utility in immune-enhancing strategies for sustainable aquaculture.

## Simple Summary

Fish are constantly exposed to various pathogens in their aquatic environment and rely heavily on innate immune responses for early defense. Cytokines are key regulators of these responses. *Tumor necrosis factor alpha* (*TNFα*) is a well-known pro-inflammatory cytokine that plays essential roles in immune modulation. In this study, we identified and characterized the *TNFα* gene in the starry flounder (*Platichthys stellatus*), a commercially important flatfish. We analyzed its gene structure, tissue-specific expression, and response to bacterial infection. Furthermore, we produced recombinant TNFα protein (rTNFα) and evaluated its immune activity in vitro. The results showed that rTNFα enhanced phagocytic activity in leukocytes without causing cytotoxic effects, supporting its potential as a molecular adjuvant in fish vaccines. These findings provide useful insights into the immune functions of TNFα and suggest its application in improving disease resistance in aquaculture species.

## 1. Introduction

Aquatic organisms are continuously exposed to diverse microbial threats in their environment, requiring robust immune mechanisms to maintain homeostasis and prevent infection. In teleost fish, the immune system comprises both innate and adaptive components, with cytokines serving as key molecular messengers that coordinate immune responses [1]. These small proteins mediate communication between immune cells, regulate inflammation, and orchestrate the activation, migration, and differentiation of leukocytes during pathogen exposure [2].

Among the various cytokines, *tumor necrosis factor alpha* (*TNFα*) has emerged as a central pro-inflammatory mediator in both mammals and fish. It is known to induce programmed cell death, stimulate inflammatory cytokine cascades, and recruit innate immune cells to infection sites [3]. In teleosts, *TNFα* has been reported to enhance leukocyte activation, promote granulocyte infiltration, and modulate downstream signaling pathways essential for pathogen elimination [4]. Studies in species such as zebrafish (*Danio rerio*) [5], rainbow trout (*Oncorhynchus mykiss*) [6], and olive flounder (*Paralichthys olivaceus*) have demonstrated that *TNFα* expression is induced upon bacterial and viral challenges in a tissue- and time-specific manner [7,8]. While *TNFα* has been investigated in olive flounder, functional studies remain limited in other flatfish species, including the starry flounder, particularly regarding its biological role and potential biotechnological applications.

Beyond its role in immune regulation, *TNFα* has recently gained attention as a potential molecular adjuvant—a biologically active agent capable of enhancing vaccine-induced immune responses when co-administered with antigens [9]. Unlike traditional adjuvants such as mineral salts or emulsified oils, cytokine-based adjuvants provide immunological specificity by directly activating antigen-presenting cells and upregulating co-stimulatory molecules, which are essential for adaptive immunity [10]. This immunostimulatory approach is of particular interest in aquaculture, where the development of effective vaccines is critical for reducing antibiotic use and controlling emerging infectious diseases.

To address these knowledge gaps, the present study focuses on the molecular and functional characterization of *TNFα* in the starry flounder, identified via transcriptomic analysis. We cloned the full-length cDNA of *TNFα*, produced recombinant protein using a cell-free expression system, and validated its biological activity through expression profiling, phagocytosis assays, and cytotoxicity evaluation. The aim of this study is to assess the immunological potential of recombinant TNFα (rTNFα) as a safe and effective molecular adjuvant in the starry flounder. The results are expected to support cytokine-based vaccine strategies and contribute to sustainable disease-management practices in aquaculture.

## 2. Materials and Methods

### 2.1. Ethics Statement, Experimental Animals, and Bacterial Strain

All experimental procedures involving animals were carried out in accordance with national ethical guidelines of South Korea and were approved by the Institutional Animal Care and Use Committee (IACUC) of the College of Marine Science, Gyeongsang National University (Approval No. GNU-241104-E0206; approved on 4 November 2024).

A total of 100 healthy starry flounders were obtained from a commercial aquaculture facility located in Pohang, Gyeongsangbuk-do, Republic of Korea. Fish were maintained in indoor recirculating seawater systems for a 2-week acclimation period and then held at a temperature of 25 ± 1 °C. Fish had an average total length of 21.5 ± 1.3 cm and an average body weight of 128.7 ± 18.2 g.

Prior to bacterial challenge, three individuals were randomly selected and examined for external and internal pathological signs to confirm their suitability for experimentation. The *Streptococcus parauberis* strain PH0710 used in this study was obtained with official approval from the National Institute of Fisheries Science (Busan, Republic of Korea). Identification was performed by sequencing the 16S rRNA gene using the universal primer 27F (5′-AGAGTTTGATCMTGGCTCAG-3′), and sequence similarity was verified via BLAST (http://www.ncbi.nlm.nih.gov/blast (accessed on 19 June 2025)) analysis in the NCBI database. A GenBank accession number is currently unavailable for this strain. Bacterial cultures were grown in Brain Heart Infusion (BHI) broth at 27 °C for 24 h, followed by centrifugation at 3000× *g* for 10 min. The harvested cells were washed twice with sterile PBS, and the final concentration was adjusted to 1 × 10^7^ CFU/mL, based on OD_600_ measurements and confirmed through plate counting.

### 2.2. Sequence Validation and Gene Characterization of TNFα in the Starry Flounder

#### 2.2.1. Cloning and Sequence Verification

The full-length open reading frame (ORF) of *TNFα* identified via NGS-based transcriptome analysis was validated by TA cloning and Sanger sequencing. Gene-specific primers were designed based on the terminal ORF sequences. PCR amplification was conducted using ExPrime Taq Premix (2×) (Genetbio, Daejeon, Republic of Korea) in a 20 µL reaction containing 10 µL of premix, 1 µL of each primer, 1 µL of cDNA template, and 7 µL of distilled water. Amplified products were electrophoresed on a 1.2% agarose gel containing 0.01% SafeView Classic (abm, New York, NY, USA), and target bands were extracted using the QIAquick Gel Extraction Kit (Qiagen, Hilden, Germany).

Purified PCR products were ligated into the pGEM-T Easy Vector (Promega, Madison, WI, USA) and transformed into *E. coli* JM109 competent cells via heat shock. Following incubation in SOC medium at 37 °C for 4 h, the transformed cells were plated on LB agar supplemented with ampicillin, X-gal, and IPTG. Positive colonies were selected and cultured overnight in LB broth. Plasmids were extracted using the Hybrid-Q™ Plasmid Rapidprep Kit (GeneAll, Seoul, Republic of Korea), and plasmids were sequenced commercially (Bioneer, Daejeon, Republic of Korea).

#### 2.2.2. Analysis and Phylogenetic Classification

The verified *TNFα* sequence was translated into an amino-acid sequence using GENETYX v8.0 (SDC Software Development, Tokyo, Japan). Homology searches were performed with BLAST against the NCBI protein database. Conserved domains and motifs were identified via the PROSITE tool and SMART database. Multiple sequence alignment was conducted using ClustalX2, and alignments were visualized in GeneDoc (version 2.7). A phylogenetic tree was constructed with MEGA v4.0 using the neighbor-joining method and 1000 bootstrap replicates to assess node reliability.

### 2.3. Expression Analysis of TNFα in the Starry Flounder

#### 2.3.1. RNA Extraction and cDNA Synthesis

To evaluate tissue-specific and infection-induced expression of *TNFα*, total RNA was extracted from 12 tissues (brain, eye, gill, head kidney, heart, intestine, liver, muscle, skin, spleen, stomach, and trunk kidney) and blood-derived leukocytes from healthy starry flounder (*n* = 5). Peripheral blood was collected via caudal venipuncture and fractionated using a 53% Percoll gradient centrifugation to isolate leukocytes and erythrocytes.

For infection trials, fish were intraperitoneally injected with *Streptococcus parauberis* PH0710 (1 × 10^3^ CFU/fish) suspended in PBS and maintained at 25 ± 1 °C. *S. parauberis* strain PH0710 was cultured in 15 mL of Brain Heart Infusion (BHI) broth (BD Difco™, Franklin Lakes, NJ, USA) at 27 °C for 24 h under constant agitation (200 rpm). Bacterial growth was monitored by measuring the optical density at 595 nm (OD_595_) and adjusted to 0.15, which corresponded to approximately 1 × 10^7^ CFU/mL based on plate count validation. The culture was subsequently washed twice with sterile PBS and diluted to the target concentration of 1 × 10^3^ CFU per 100 µL for injection. Tissues (brain, gill, heart, head kidney, liver, intestine, and spleen) were sampled at 0, 1, and 12 h, and at days 1, 3, 5, and 7 post-infection. All samples were stored at −80 °C until RNA isolation.

RNA extraction was carried out using RNAiso Plus (Takara, San Jose, CA, USA), followed by chloroform and PCI (phenol/chloroform/isoamyl alcohol, 25:24:1) phase separation. Genomic DNA was removed using recombinant DNase I (Takara, San Jose, CA, USA), and RNA was precipitated with isopropanol and sodium acetate. The final pellet was washed with 75% ethanol, dried, and resuspended in DEPC-treated water.

First-strand cDNA synthesis was performed using the PrimeScript™ 1st Strand cDNA Synthesis Kit (Takara, San Jose, CA, USA). Each 20 µL reaction included total RNA, random primers, dNTPs, reverse transcriptase, and RNase inhibitor, and followed the manufacturer’s protocol.

#### 2.3.2. Real-Time PCR

Expression levels of *TNFα* were measured by SYBR Green-based real-time PCR using TB Green™ Premix Ex Taq™ (Tli RNaseH Plus) (Takara, San Jose, CA, USA). Each 25 µL reaction consisted of 12.5 µL of 2X premix, 1 µL each of forward and reverse primers (10 pmol), 1 µL of cDNA, and sterile distilled water. PCR was performed on a Thermal Cycler Dice^®^ Real Time System III (Takara, San Jose, CA, USA) with the following conditions: 50 °C for 4 min, 95 °C for 10 min, followed by 45 cycles of 95 °C for 15 s and 60 °C for 30 s.

The elongation factor 1-alpha (EF-1α) gene was used as an internal control, and relative expression levels were calculated using the 2^−ΔΔCt^ method [11]. Primer sequences are provided in Table 1.

### 2.4. Production of Recombinant TNFα Protein

Recombinant TNFα protein was produced using a cell-free protein synthesis system (ExiProgen; Bioneer, Daejeon, Republic of Korea) in accordance with the manufacturer’s instructions. The *TNFα* gene was inserted into the pBIVT-2 expression vector containing a 6×His tag to facilitate purification. Protein synthesis was carried out at 30 °C in a reaction mixture comprising *E. coli* lysate supplemented with T7 RNA polymerase, ribosomes, tRNAs, amino acids, and an energy-regenerating system.

Following expression, the recombinant protein was purified using Ni-affinity chromatography and stored in a stabilization buffer (50 mM Tris-Cl, pH 7.6; 100 mM NaCl; 1 mM DTT; 0.1 mM EDTA; 0.05% NaN_3_; and 50% glycerol). Protein purity and molecular size were assessed via 12% SDS-PAGE, and concentration was quantified using the Bradford method with a commercial kit (Bio-Rad, Hercules, CA, USA).

### 2.5. Functional Evaluation of Recombinant TNFα as a Molecular Adjuvant

#### 2.5.1. Phagocytic Activity Assay

Leukocytes were isolated from the kidney, spleen, and gill tissues of starry flounder using a 53% Percoll solution. The Percoll mixture was prepared by combining 26.5 mL of Percoll with 2.95 mL of 10× PBS and 20.55 mL of PBS. Tissues were passed through 100 µm strainers with 2 mL of RPMI medium (Sigma-Aldrich, Burlington, MA, USA), and cell suspensions were layered onto the 53% Percoll and centrifuged at 500× *g* for 15 min at 20 °C. The leukocyte layer was collected, washed twice with PBS, and erythrocytes were lysed with Red Blood Cell Lysing Buffer Hybri-Max (Sigma-Aldrich, Burlington, MA, USA). Final leukocyte suspensions were adjusted to 1 × 10^6^ cells/mL.

Phagocytic activity was evaluated using *S. parauberis* PH0710 labeled with fluorescein isothiocyanate (FITC, 10 mg/mL in DMSO). Leukocytes were treated with FITC-*S. parauberis* in the presence of rTNFα at concentrations of 50, 100, 150, or 200 µg/mL. Control cells received only FITC-*S. parauberis* and PBS. All samples were incubated for 30 min at room temperature in the dark.

#### 2.5.2. Hemolytic Activity Assay

Erythrocytes were isolated as described in Section 2.5.1 and resuspended in PBS at a 4% concentration. rTNFα was diluted to six final concentrations (1, 10, 50, 100, 150, and 200 µg/mL). In a 96-well plate, 50 µL of erythrocyte suspension was mixed with 50 µL of each protein dilution. Positive controls were treated with 0.1% Triton X-100, and negative controls received PBS only. Following incubation at room temperature for 30 min, samples were centrifuged at 1000× *g* for 10 min. Supernatants were transferred to a fresh plate, and absorbance was measured at 540 nm to quantify hemolytic activity.

#### 2.5.3. Immune Induction by Molecular Adjuvants at Different Concentrations

To examine the time-dependent mRNA expression levels in the tissues of starry flounder stimulated with molecular adjuvants, rTNF protein was intraperitoneally injected into healthy fish at concentrations of 50, 100, and 150 μg/m. The fish were maintained at a water temperature of 15 ± 1 °C. At 0, 1, and 12 h and on days 1, 3, 5, and 7 post-injection, three fish were randomly selected, and their kidneys were excised. The tissues were stored at −80 °C until total RNA extraction. All protocols from RNA extraction to cDNA synthesis were performed as described in Section 2.3.1.

mRNA expression levels of specific genes were measured by quantitative real-time PCR (RT-qPCR) with TB Green™ Premix Ex Taq™ (Takara, San Jose, CA, USA) using the SYBR Green method. The PCR reaction mixture consisted of 12.5 µL of TB Green Premix Ex Taq 2X (Tli RNaseH Plus, Takara Bio, San Jose, CA, USA), 1 µL each of the forward and reverse primers (10 pmol), 1 µL of cDNA template, and sterilized distilled water to a final volume of 25 µL. The specific primers used for the RT-qPCR analysis, including IL-1β, TNF, and EF-1α, along with their sequences, are provided below. PCR was performed using the Thermal Cycler Dice^®^ Real-Time System III (Takara, San Jose, CA, USA) with the following conditions: 4 min at 50 °C, 10 min at 95 °C, followed by 45 cycles of 15 s at 95 °C and 30 s at 60 °C. The cycle threshold (Ct) values were normalized to those of elongation factor 1-alpha (EF-1α) using the delta delta Ct method. The specific primers used for the RT-qPCR analysis were as follows: *IL-1β* (forward: 5′-TGC AGT GGT CAA GAT GAT GGA-3′, reverse: 5′-AGT TGC GGT GAA GTC AAG CAG-3′), *TNF* (forward: 5′-ATC TGG AGA CCC ACG ACT GT-3′, reverse: 5′-TCC AGA AGA GGA TGT CGG TT-3′), and *EF-1α* (forward: 5′-TGA CGA GAT CGA GAA GTC CA-3′, reverse: 5′-GAC ATT GTC ACC AGG GAG GT-3′).

### 2.6. Statistical Analysis

All experiments were conducted in triplicate, and data are presented as mean ± standard deviation (SD). Statistical significance was determined by one-way analysis of variance (ANOVA), followed by Tukey’s post hoc test using SPSS version 19 (IBM, Armonk, NY, USA). Differences were considered statistically significant at * p < 0.05 and highly significant at ** p < 0.01.

## 3. Results

### 3.1. Identification and Sequence Characteristics of TNFα in the Starry Flounder

The open reading frame (ORF) of the *TNFα* gene from the starry flounder comprised 771 base pairs, encoding a protein of 257 amino acids. Sequence analysis identified a transmembrane region (amino acids 35–57) and a conserved TNF domain (amino acids 95–256). The validated sequence has been deposited in the NCBI GenBank database under accession number PQ565689 (Table 2).

**Figure 1:**
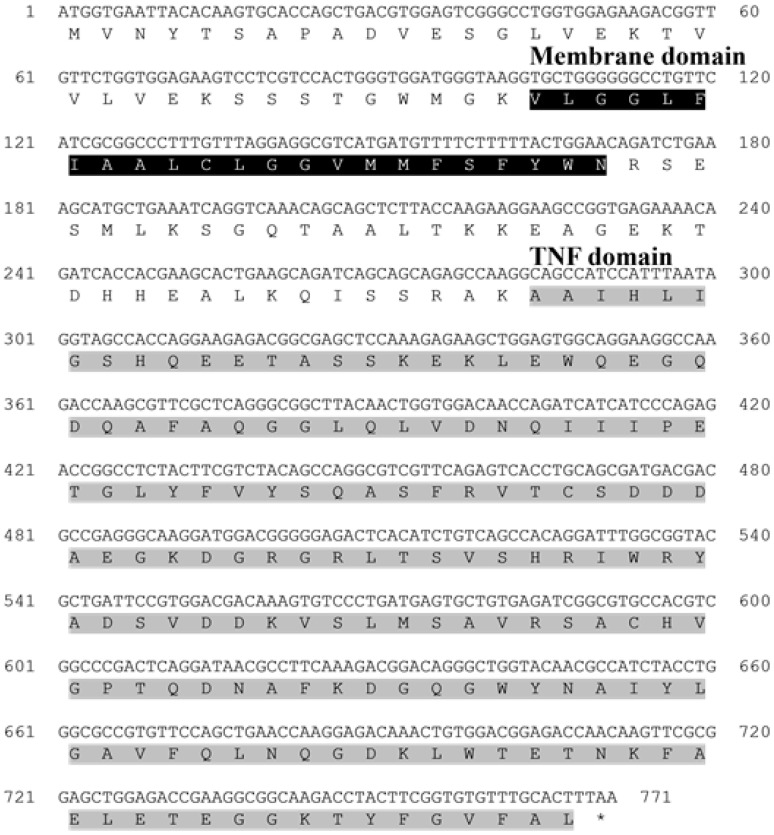
Nucleotide and deduced amino acid sequences of *TNFα* from the starry flounder. The gray boxes indicate a TNF domain, while the black box represents the membrane domain. The asterisk (*) indicates the stop codon in the amino acid sequence.

**Figure 2:**
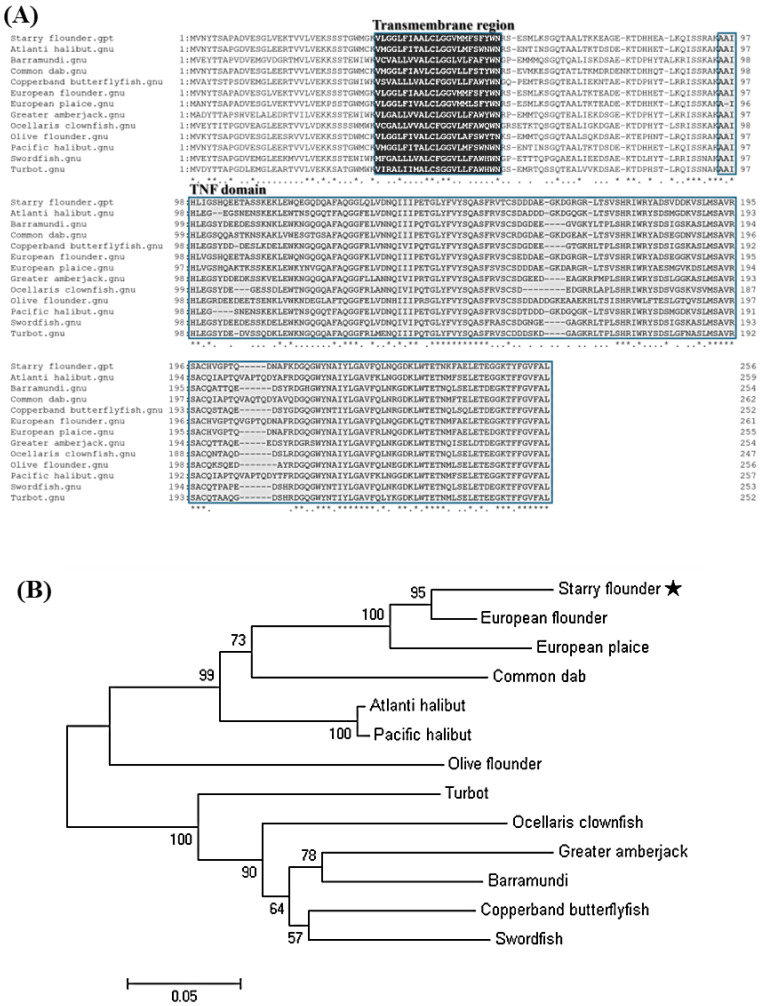
(**A**) Multiple sequence alignment of the predicted *TNFα* amino acid sequence from the starry flounder with *TNFα* sequences from other species. The conserved TNF domain is highlighted in gray, and the transmembrane region is indicated in black. Asterisks (*) indicate fully conserved residues among the aligned sequences. (**B**) Phylogenetic tree constructed using the neighbor-joining (NJ) method, showing the relationship between starry flounder *TNFα* and its homologs from other species. Bootstrap values (based on 1000 replicates) are shown at branch nodes, and the scale bar represents a branch length of 0.05. The star (★) indicates the starry flounder used in this study.

Amino-acid sequence alignment demonstrated that *TNFα* from the starry flounder shares a high degree of conservation with homologs from other teleosts, exhibiting sequence identities ranging from 63% to 91.57% (Table 2; Figure 1). Phylogenetic analysis further positioned the starry flounder *TNFα* within a clade of marine fish *TNFα* sequences, showing the closest evolutionary relationship with that of the European flounder (Figure 2A,B).

### 3.2. Expression Profiles of TNFα in the Starry Flounder

**Figure 3:**
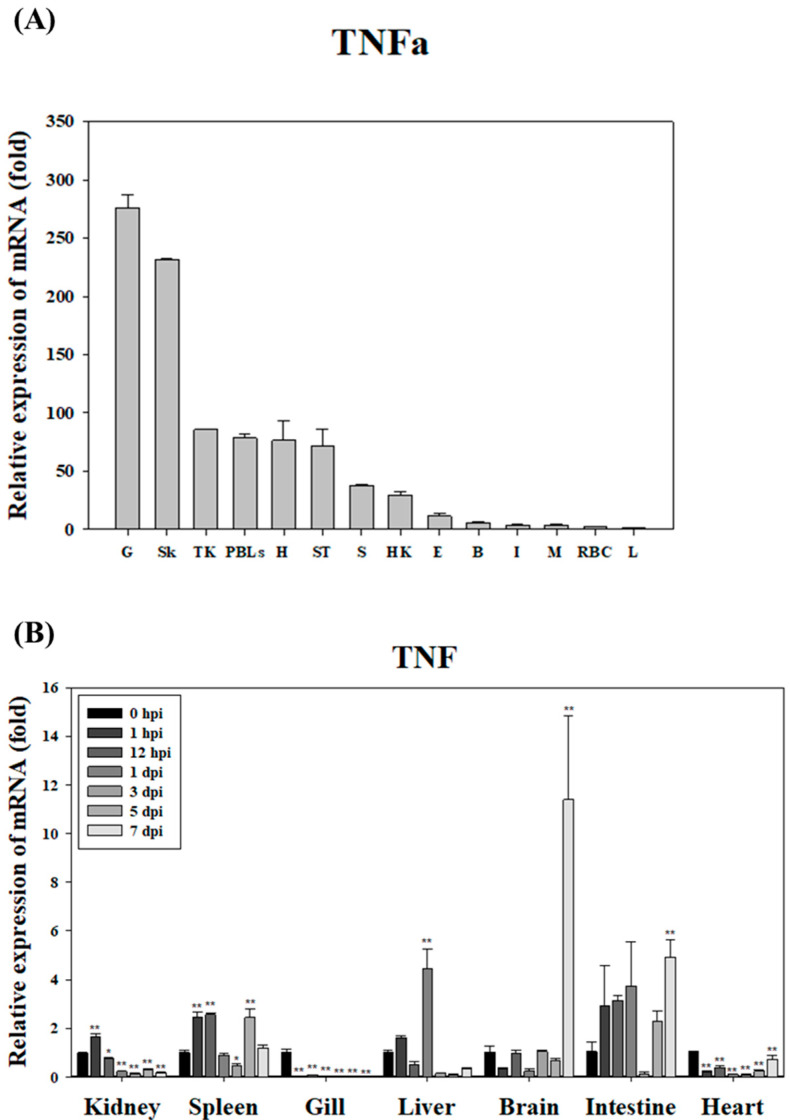
Relative expression levels of TNFα mRNA in starry flounder tissues. (**A**) Basal expression of TNFα in various tissues of healthy starry flounder. Expression levels were normalized to EF-1α and are presented as fold changes relative to the liver. Data represent the mean ± SD from five independent cDNA samples, each analyzed in triplicate. (**B**) Temporal expression of TNFα mRNA in the brain, gill, heart, head kidney, liver, intestine, and spleen following *S. parauberis* infection. Values are shown as mean ± SD. Asterisks indicate statistically significant differences compared to the control (0 h) (* *p* < 0.05, ** *p* < 0.01).

In healthy starry flounder, *TNFα* mRNA expression was highest in the gills, exhibiting a 276-fold increase relative to the liver, which showed the lowest expression. Elevated levels were also detected in the skin (232-fold) and kidney (86-fold), whereas relatively low expression was observed in the red blood cells (RBCs), muscle, and intestine (Figure 3A). Following infection with *S. parauberis* PH0710, *TNFα* expression was significantly downregulated in the gills and heart throughout most of the infection period. In contrast, a marked upregulation was observed in the brain and intestine, with the brain displaying peak *TNFα* expression on day 7 post-infection. Additionally, significant early-phase upregulation was observed in the kidney and spleen (Figure 3B).

### 3.3. Confirmation of Recombinant TNFα Production

**Figure 4:**
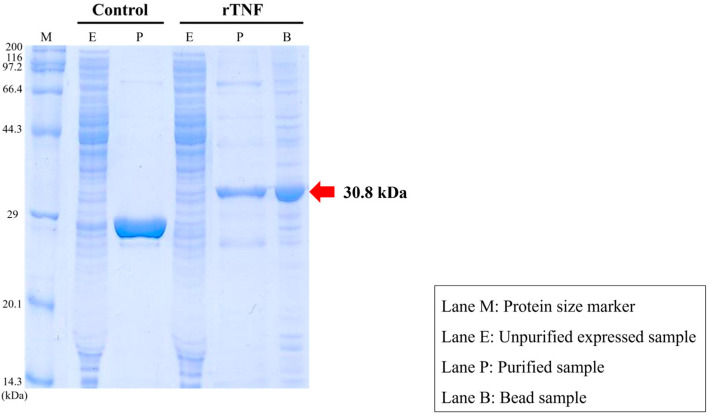
12% SDS-PAGE analysis of TNFα recombinant protein. Control: AcGFP is a basic (constitutively fluorescent) green fluorescent protein, derived from *Aequorea coerulescens*. Lane M: molecular weight marker; Lane E: crude extract of expressed rTNFα; Lane P: purified rTNFα protein used for functional assays; Lane B: bead-bound fraction after purification. The AcGFP control was used independently to verify the performance of the cell-free expression and purification system, but it was not used in any functional assay.

Recombinant TNFα protein (rTNFα) was produced to evaluate its potential as a molecular adjuvant for the S. parauberis formalin-killed cell (FKC) vaccine. The predicted molecular weight of rTNFα, including the ~2.7 kDa 6 × His tag, was approximately 28.05 kDa, which corresponded to the observed band in the SDS-PAGE analysis (Figure 4). The successful expression and approximate size of the recombinant protein were confirmed by 12% SDS-PAGE and further validated by Western blot analysis using an anti-His tag antibody (Supplementary Figure S1).

### 3.4. Phagocytic Activity of Recombinant TNFα Protein

The immunostimulatory effect of recombinant TNFα protein (rTNFα) on phagocytic activity was evaluated in peripheral blood leukocytes (PBLs) and trunk kidney leukocytes from starry flounder. Gating analysis was performed to identify target leukocyte populations, and phagocytic activity was assessed following stimulation with rTNFα.

**Figure 5:**
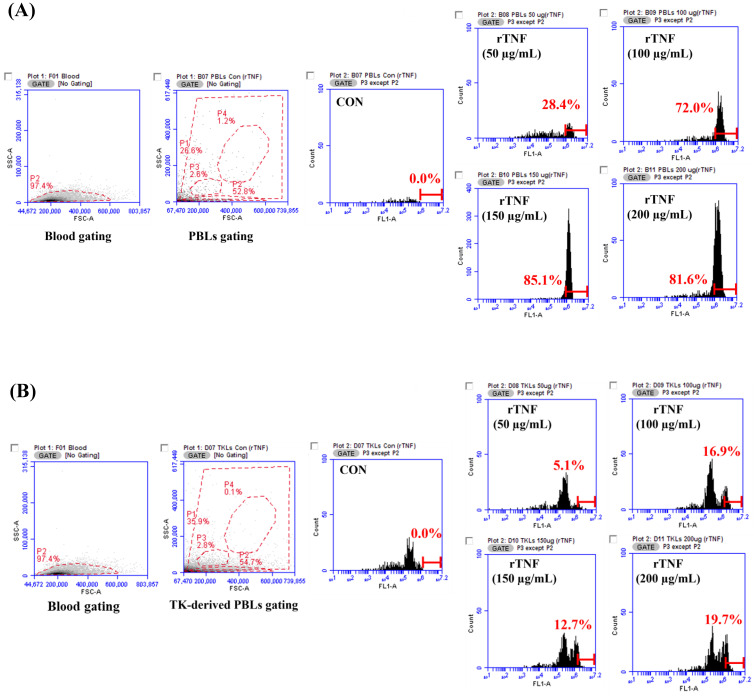
Flow cytometric analysis of phagocytic activity in peripheral blood leukocytes (PBLs) and trunk kidney leukocytes following treatment with recombinant TNFα (rTNFα). (**A**) PBLs were gated based on FSC/SSC parameters (left panel), and phagocytic activity was assessed after stimulation with different concentrations of rTNFα. (**B**) Trunk kidney leukocytes were similarly gated (left panel), and phagocytic activity was evaluated following rTNFα treatment.

In PBLs, phagocytic activity significantly increased in a concentration-dependent manner, reaching 28.4% at 50 μg/mL, 72.0% at 100 μg/mL, and peaking at 85.1% at 150 μg/mL, followed by a slight decline to 81.6% at 200 μg/mL (Figure 5A).

In trunk kidney leukocytes, activity increased to 5.1% at 50 μg/mL and 16.9% at 100 μg/mL, peaked at 19.7% at 200 μg/mL, but dipped to 12.7% at 150 μg/mL (Figure 5B).

### 3.5. Cytotoxicity of Recombinant TNFα Protein

**Figure 6:**
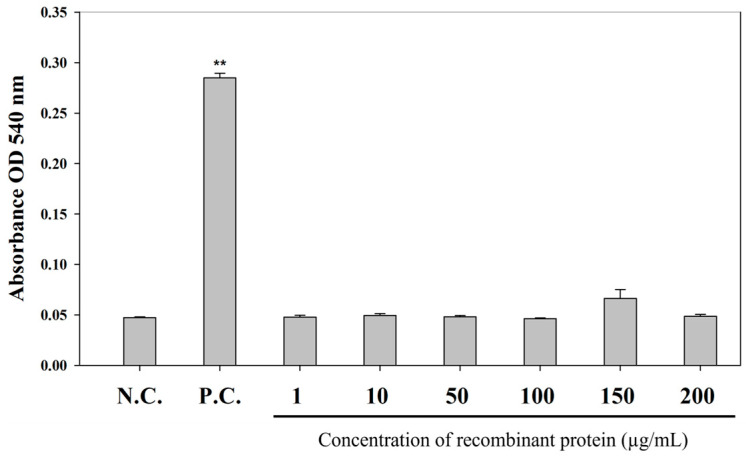
The hemolytic and cytotoxicity of rTNFα, analyzed using starry flounder erythrocytes. The positive control was measured by adding a 0.1% Triton X-100 solution. Values are expressed as the mean ± SD, and asterisks indicate a significant difference (** *p* < 0.01) from the negative control (PBS buffer).

To assess the in vivo safety of recombinant TNFα (rTNFα), a hemolysis assay was conducted using erythrocytes isolated from starry flounder. No hemolytic activity was observed in any of the rTNFα-treated groups, even at the highest concentration of 200 μg/mL, and the results were comparable to those of the PBS-treated negative control group. In contrast, hemolysis was evident in the positive control group (Figure 6).

### 3.6. Immune-Inducing Capacity of rTNFα

To evaluate the immune-inducing capacity of the molecular adjuvant rTNF, total RNA was extracted from major tissues of the starry flounder following rTNF administration. The RNA was converted into cDNA, and mRNA expression levels of immune-related genes were quantified using real-time PCR.

**Figure 7:**
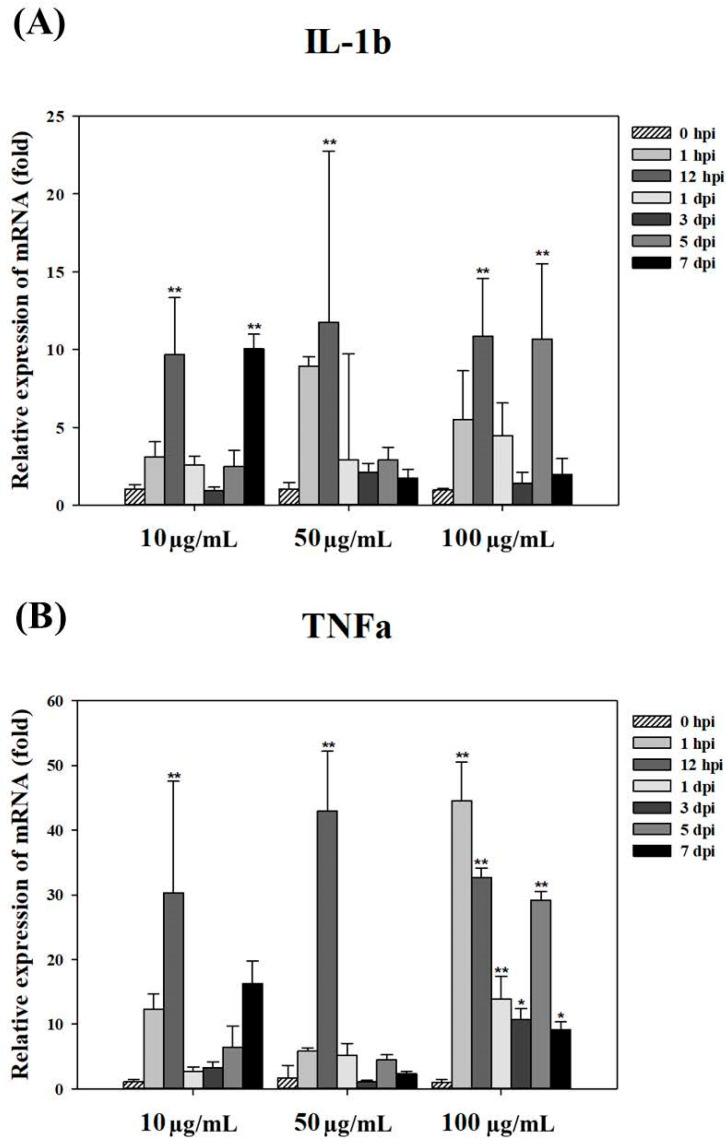
(**A**) Expression levels of *IL-1β* mRNA in the kidney of starry flounder following stimulation with varying concentrations of rTNFα. Gene expression levels are presented as the mean ± SD. Asterisks indicate statistically significant differences compared to the control group (0 h) (* *p* < 0.05, ** *p* < 0.01). (**B**) Expression levels of *TNFα* mRNA in the kidney of starry flounder following stimulation with varying concentrations of rTNFα. Gene expression levels are presented as the mean ± SD. Asterisks indicate statistically significant differences compared to the control group (0 h) (* *p* < 0.05, ** *p* < 0.01).

After stimulation with rTNFα at concentrations of 10, 50, and 100 μg/mL, a significant increase in IL-1β, an inflammatory cytokine, was observed at 50 μg/mL and 100 μg/mL concentrations at 1 h and 1 day post-stimulation. Notably, maximum expression (approximately 20-fold) was observed at 1 day post-stimulation at 50 μg/mL. This indicates that rTNFα strongly induces *IL-1β* expression, enhancing the inflammatory response (Figure 7A).

TNFα, a key cytokine involved in inflammation and apoptosis, exhibited significant up-regulation at all tested concentrations (10, 50, and 100 μg/mL). The maximum expression level (approximately 45-fold) was observed at 3 days post-stimulation at 50 μg/mL, while a significant increase was noted at 5 days post-stimulation at 100 μg/mL. These results suggest that rTNFα modulates *TNFα* expression in a concentration- and time-dependent manner (Figure 7B).

## 4. Discussion

In this study, *TNFα* was identified in the starry flounder through next-generation sequencing (NGS) analysis, and its sequence characteristics were investigated. Conserved domains and functional motifs were annotated using the PROSITE profile database and the Simple Modular Architecture Research Tool (SMART). Multiple sequence alignment and phylogenetic analysis were performed to infer the potential immunological roles of the identified *TNFα* gene in the starry flounder.

*TNFα* is a key pro-inflammatory cytokine involved in the regulation of acute immune responses during infection in mammals. The *PsTNFα* gene identified in this study comprised a 771 bp open reading frame (ORF) encoding 257 amino acids. Structural analysis revealed the presence of a conserved TNF domain and a predicted transmembrane region, indicating that *PsTNFα* shares the typical features of the *TNFα* family. Sequence alignment showed 91.57% identity with European flounder *TNFα*, and phylogenetic analysis clustered *PsTNFα* with homologues from other marine teleosts, suggesting evolutionary conservation. These results indicate that *PsTNFα* likely plays a central role in the modulation of inflammatory responses and innate immunity in fish.

In this study, *TNFα* expression in healthy starry flounder was found to be high in pathogen-contact tissues, such as the gills, skin, and head kidney. This suggests that *TNFα* may promote inflammatory responses at the sites of pathogen invasion, thereby contributing to early defense mechanisms. Following infection, TNFα expression decreased in the gills and heart, but increased in certain tissues, such as the brain and intestine. This indicates that *TNFα* may regulate immune responses in a tissue-specific manner. Notably, the significant increase in *TNFα* expression observed in the brain on day 7 post-infection implies that the central nervous system may play a role in regulating inflammation and controlling the spread of infection. This finding suggests that the nervous system could be an integral part of the immune system. Tissue-specific expression of *TNFα* has also been reported in white crucian carp, where recombinant TNFα1 played a crucial role in gut immune regulation following pathogen exposure, underscoring its diverse immunomodulatory functions across species [12].

These results align with previous studies showing that *TNFα* acts as a regulator of inflammation and immune responses during the early stages of infection in fish [2]. Similarly, studies have reported that *TNFα* expression in mollusks and fish is differentially regulated across tissues depending on bacterial or viral stimuli [13]. This study suggests that *TNFα* in the starry flounder may also possess mechanisms to regulate its expression in specific tissues to respond effectively to infections. Therefore, *TNFα* appears to play an important role in inflammation and early defense against infections in the starry flounder, with its tissue-specific expression representing a strategic mechanism for localized immune responses.

In the present study, we successfully produced and characterized rTNFα derived from starry flounder and confirmed its immunological activity through a series of functional assays. Functionally, rTNFα demonstrated a concentration-dependent enhancement of phagocytic activity in both peripheral blood leukocytes (PBLs) and trunk kidney leukocytes. Maximal activation occurred at 150 μg/mL, supporting the notion that TNFα serves as a key regulator of innate immune responses in fish. Interestingly, PBLs and trunk kidney leukocytes showed slightly different response patterns, possibly due to differences in cell composition and activation thresholds, as trunk kidney leukocytes include a more heterogeneous population, including hematopoietic precursors. These findings are consistent with previous studies reporting that recombinant TNFα enhances phagocytic function in various teleost species [14,15,16,17,18]. For example, in the rainbow trout (*Oncorhynchus mykiss*), *TNFα* stimulation increased macrophage respiratory burst and phagocytosis, while in the zebrafish (*Danio rerio*), recombinant TNFα induced inflammatory gene expression and phagocytic activation in macrophage-like cells [16]. These parallels suggest that the enhanced phagocytosis observed in our study reflects conserved biological activity of *TNFα* across fish species. The immune-enhancing effect of rTNFα is likely mediated through *TNF* receptor signaling pathways such as *TNFR1* and *TNFR2*, which activate downstream effectors, including NF-κB and MAPKs, leading to increased immune-cell activation and antimicrobial activity. In our study, phagocytic activity slightly declined at the highest concentration (200 μg/mL), suggesting a possible feedback inhibition or functional exhaustion, as previously observed with other immunostimulants such as levamisole and β-glucans [19,20,21]. This highlights the importance of optimizing cytokine dosage to maximize immunostimulatory effects while avoiding overstimulation. Overall, our results demonstrate that rTNFα can effectively activate innate immune cells in vitro without inducing cytotoxicity, as confirmed by hemolysis assays. These findings support the potential utility of rTNFα as an immunostimulant in aquaculture, particularly in vaccine adjuvant strategies aimed at enhancing early-phase immune responses and reducing reliance on antibiotics.

The observed upregulation of *IL-1β* and *TNF* mRNA following rTNFα stimulation confirms the immunostimulatory potential of the recombinant protein in the starry flounder. IL-1β is a key pro-inflammatory cytokine that plays a central role in initiating innate immune responses, while TNF is involved in amplifying inflammatory signaling and regulating apoptosis. The significant induction of both genes at early time points suggests that rTNFα effectively activates inflammatory pathways, thereby enhancing the host’s ability to respond to pathogenic threats. These findings support the hypothesis that rTNFα can serve as a functional molecular adjuvant by modulating key immune mediators in fish.

Collectively, these findings suggest that *TNFα*, beyond its canonical pro-inflammatory role, holds promise as a molecular adjuvant in fish immunology.

Its ability to boost phagocytic activity, paired with a favorable safety profile, supports its application in the design of next-generation vaccines. Notably, recombinant TNFα has also been shown to confer protective effects as an oral vaccine adjuvant in the European sea bass, where it enhanced mucosal immune responses against *Vibrio* infection through modulation of the CCL25/CCR9 axis [22]. Unlike traditional adjuvants, cytokines like *TNFα* may offer precise immunological tuning with minimal off-target effects.

Future work should expand toward in vivo validation and field-based trials to assess its adjuvant efficacy under commercial aquaculture conditions. The foundational insights provided by this study may inform cytokine engineering strategies and contribute to sustainable fish health management by reducing antibiotic dependency.

To account for the possibility of nonspecific immune stimulation, the recombinant rTNFα protein used in this study was purified through Triton X-114 phase separation, a method widely used to remove endotoxin contamination. Although a direct quantification of endotoxin levels (e.g., via LAL assay) was not performed, several lines of evidence support the biological activity of rTNFα as the primary cause of the observed immune responses. The purified protein was confirmed by SDS-PAGE and Western blotting, and it induced a concentration-dependent increase in phagocytic activity, which aligns with the known functional role of TNFα in immune-cell activation. Moreover, the hemolysis assay confirmed the absence of cytotoxicity, indicating that the immune effects were not caused by nonspecific cell damage. Nevertheless, we recognize the importance of further validating these results through the inclusion of endotoxin-only controls in future studies.

## 5. Conclusions

In summary, this study characterized the molecular structure and immunological function of *TNFα* in the starry flounder and evaluated its potential as a molecular adjuvant. The *PsTNFα* gene exhibited conserved domain structures and phylogenetic relatedness to *TNFα* from other marine fish species. Expression profiling revealed its tissue-specific regulation during infection, particularly highlighting immune-neural interactions. Recombinant TNFα significantly enhanced phagocytic activity in leukocytes in a dose-dependent manner, while maintaining safety with no observed hemolytic activity. These findings support the potential of rTNFα as a safe and effective molecular adjuvant for improving vaccine efficacy in aquaculture species. Further research is warranted to optimize dosing strategies and evaluate its synergistic effects with other immunostimulants under practical aquaculture conditions.

## Figures and Tables

**Table 1:** List of primer sets used for RT-qPCR.

Usage	Primer	Primer Sequence (5′-3′)	Efficiency (%)	R^2^	Pearson’s r
Housekeeping gene	*EF-1α* (F)	GTGGCAAGTCCACCACCA	97.2	0.998	0.998
	*EF-1α* (R)	GCTTGTCCAGCACCCAGG			
Target gene	*TNFα* (F)	CAAAGAGAAGCTGGAGTGGC	94.5	0.997	0.996
	*TNFα* (R)	TGGTGAGGTCTCTGATGTCG			

F = forward primer, R = reverse primer, Efficiency (%) = qPCR amplification efficiency, R^2^ = coefficient of determination, Pearson’s r = Pearson correlation coefficient.

**Table 2:** Sequence homology (%) of *TNFα* between the starry flounder and other species.

Species	Domain Length (aa)	GenBank Accession Number	Sequence Homology
Starry flounder	161	PQ565689	-
European flounder	167	XP_062254352.1	91.57%
European plaice	162	XP_053288649.1	86.72%
Atlantic halibut	165	XP_034465774.1	77.78%
Common dab	167	XP_060937363.1	77.48%
Pacific halibut	163	XP_035019705.1	77.39%
Barramundi	159	XP_018555325.1	67.32%
Olive flounder	162	XP_019960518.1	67.05%
Ocellaris clownfish	152	XP_023141970.1	65.37%
Turbot	158	XP_035485753.1	65.37%
Swordfish	159	XP_039973827.1	65.23%
Copperband butterflyfish	185	XP_041797645.1	64.84%
Greater amberjack	160	XP_022602098.1	63.57%

## Data Availability

The original contributions presented in this study are included in the article/Supplementary Materials. Further inquiries can be directed to the corresponding author.

## Supplementary Materials

The following supporting information can be downloaded at: https://www.mdpi.com/article/10.3390/ani15142119/s1, Figure S1: Western blot analysis of rTNFα. Lane M: marker; Lane 1: purified TNFa recombinant protein.
