# Supplementary figures and images for: Functional Characterization of TNFα in the Starry Flounder (Platichthys stellatus) and Its Potential as an Immunostimulant
Source: Animals (Basel). 2025 Jul 17;15(14):2119. doi: 10.3390/ani15142119 (PMC12291946; doi:10.3390/ani15142119)

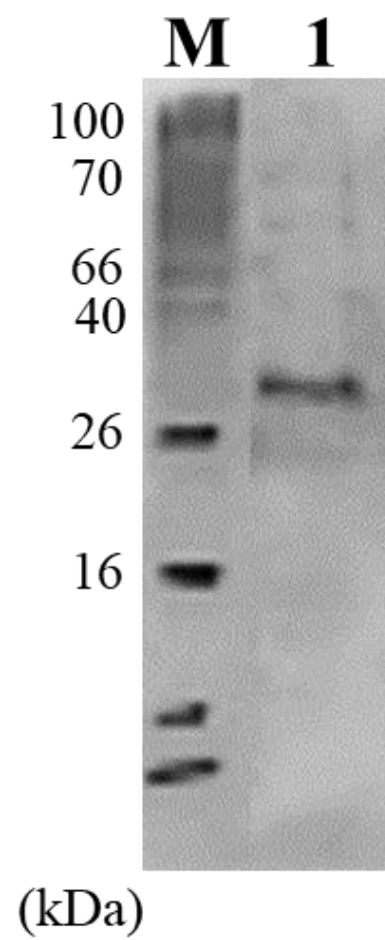

Supplementary Figure S1. Sohn et al.

Supplement: Supplementary file 1 [file animals-15-02119-s001.zip › animals-3706984-supplementary.pdf]
